# Influence of risk literacy, decision-making styles and motivation on clinical reasoning in medical students: an ordinal logistic regression analysis

**DOI:** 10.1186/s12909-025-07135-5

**Published:** 2025-04-29

**Authors:** Hamsa Al-Sayyed, Felix Albert, Eva Schönefeld, Roman-Patrik Lukas, Hendrik Friederichs

**Affiliations:** 1 Department of Anesthesiology, Intensive Care and Pain Medicine, University Hospital Münster, Albert- Schweitzer-Campus 1, 48149 Münster, Germany; 2 Institute of Biostatistics and Clinical Research, University of Münster, Schmeddingstr. 56, 48149 Münster, Germany; 3 Institute of Medical Education, Medical Faculty of Münster, Niels-Stensen-Str. 12, 48149 Münster, Germany; 4 Medical Education Research Group, Medical School OWL, Bielefeld University, Bielefeld, Germany

**Keywords:** Medical education, Clinical reasoning, Risk literacy, Decision-making styles, Bayes’ Theorem

## Abstract

**Background:**

Clinical reasoning is critical to the medical profession and should be a central component of the medical curriculum. However, there are different explanations of how clinical reasoning works, and there is little research on how it develops during medical education. The aim of this study is to investigate factors, i.e. skills, processes and motivations, which influence clinical reasoning in medical students.

**Methods:**

128 data sets were included in our study.

We focused on the diagnostic aspect and therefore used students’ Bayesian reasoning ability in three medical case scenarios (0–3 points) as the outcome parameter.

The study measures students’ risk literacy (Berlin Numeracy Test, 0–4 points) as a skill, their decision-making style as an intuitive and/or rational process (Decision Styles Scale, each 1–5 points) and identifies the role of motivation (questionnaire with 5-point Likert scales) in relation to academic goals as a potential influence on clinical reasoning.

We used an ordinal logistic regression model for analysis.

**Results:**

Ordinal logistic regression showed that risk literacy is more important for solving medical case scenarios than students’ motivation to become researchers.

The chance of solving ≥ 1 scenario increased by 33% when the highest BNT score was compared to the lowest, compared to a 27% increase for the highest motivation to become a researcher. The probability of obtaining ≥ 1 point in the scenarios rose 20% when the BNT score went from three to four, indicating that highly risk-literate students have a higher ability to solve Bayesian tasks. Yet, the ability to perform Bayesian reasoning also increased consistently with growing motivation to become a researcher.

**Conclusions:**

The study highlights the importance of paying attention to medical students’ risk literacy in the development of clinical reasoning, as it appears to be a critical component. Motivation also plays an important role and, accordingly, should be encouraged in medical education.

**Supplementary Information:**

The online version contains supplementary material available at 10.1186/s12909-025-07135-5.

## Background

In a medical context, clinical reasoning has been identified as a core competency and is required in the toolbox of every physician [1]. Hence, the medical curriculum should explicitly address this competency and nurture it in medical students [2]. However, there are many conceptions of clinical reasoning, as it is a multi-dimensional construct and has no clear terminology [3]. Young et al. [4] attempted to map the literature and identified common categories of clinical reasoning definitions, including as a *skill*, i.e. the abilities necessary to make successful clinical judgements, and as a *process* describing how reasoning takes place, such as analytical versus intuitive thinking. Previous research uncovered a general scarcity on studies about clinical reasoning with available evidence often focusing on influencing contextual factors (e.g. noise, time, etc.), [5] and study populations consisting of physicians or different medical professions [5–8]. To contribute to the identification of learning objectives for the medical curriculum, this study aimed to explore factors influencing clinical reasoning in medical students.

Hence, we surveyed deliberate aspects of medical students’ traits, namely *skills* and *process*, and their *motivation* based on the assumption that if clinical reasoning can be learned, motivations must play a role [9]. Among the many subparts of clinical reasoning, diagnostic thinking is one of the most frequently mentioned, mostly placed in the category of the *outcome* or purpose of clinical reasoning [3]. Therefore, this study focused on this aspect of clinical reasoning.

Bayesian estimations are applied for diagnosing and treating a patient’s illness [10, 11]. Thus, Bayesian reasoning was used as the *outcome* parameter to measure clinical reasoning. To illustrate this process, imagine a physician who is tasked with diagnosing a patient. First, they consider a disease that might fit the patient’s symptoms. Next, they assess the disease’s pre-test probability, which is heavily influenced by prevalence. Then, they perform examinations and tests, and the results modify the post-test probability. To compute this post-test probability, Bayes’ rule is applied, using the values of the disease’s prevalence and performed tests’ sensitivity and specificity [10]. One example of this method would be the computation of positive predictive values, which can be done by dividing the true-positives (Sensitivity*Prevalence) by the sum of true-positives and false-positives (Sensitivity*Prevalence + (1-Specificity)*(1-Prevalence)). Employing this method, Bayes’ Theorem provides the mathematical groundwork for the diagnostic thinking of clinicians as it is close to the hypothetico-deductive model of clinical reasoning. In this model the physician develops hypotheses on a patient’s disease and then updates their probabilities with the arrival of new information [12]. The required numeracy can be measured as so-called risk literacy [13]. Medical students score very low when confronted with clinical vignettes that require the application of Bayes’ Theorem [14, 15], which is also true for physicians [16, 17]. However, those physicians who are able to successfully apply Bayes’ Theorem often display higher levels of risk literacy [18].

Risk literacy refers to one’s ability to “accurately interpret and act on information about risk” (p. 26) [13]. Since Bayes’ Theorem and risk literacy both necessitate probabilistic thinking, this study measured risk literacy as the *skill* required for clinical reasoning. A person’s level of risk literacy mostly relies on their statistical numeracy [19]. Although risk literacy can easily be measured by evaluating a person’s performance on mathematical tests, it does not solely predict numerical judgements, as it also predicts many non-numerical judgements, such as medical, financial, and metacognitive tasks [13, 20]. Cokely et al. [19] explored differences in decision-making between experts and non-experts and what makes a decision superior. They defined the general decision-making skill as “stable differences in judgement and decision making quality exhibited across diverse and wide-ranging domains” (p. 477) and stated that its central mechanism is statistical numeracy, which is important in decision-making contexts.

Decision-making styles are the cognitive *process* we chose to investigate as a potential influence on clinical reasoning. They address people’s mode of perceiving information and responding to a proposed problem [21], which was identified as one aspect by which clinical reasoning can be categorized [4]. This study focused on the intuitive and rational decision-making styles, since they are the most prominent and consistent styles among the available measuring instruments [22]. These two decision-making styles mainly differ in the need to either thoroughly consider decisions or rely on intuition. Traditionally, people who try to approach problems rationally prefer an analytical approach,gathering all available information, then evaluating the options before making a decision [23]. Conversely, intuitive decision-makers are mainly guided by feelings [21, 23]. Thus, questionnaires aimed at identifying this include keywords such as “hunch,” “gut feeling,” or “first impressions” [22]. Their attributes closely resemble the fast System 1 and deliberate System 2 from the dual process theory, which are both applied in the diagnostic process and can be viewed as the overarching framework [24]. In probabilistic tasks rational decision-making usually outdoes an intuitive decision style [25].

Lastly, this study identified the *motivational* aspect needed for clinical reasoning as *motivation* to achieve specific academic career goals. In the CanMEDS framework, physicians are supposed to display a wide variety of abilities, including the role of a scholar, which entails contributing to new scientific developments [26]. For this role, scholars need to develop a certain statistical and mathematical expertise to grasp scientific information which is encoded in numbers [27–29]. As Bayesian reasoning essentially relies on computational efforts, we selected aspects of this CanMEDS role as a potential influence.

### Study aims

Clinical reasoning is a multi-faceted construct made up of many different aspects. Therefore, this study focused on medical students’ Bayesian reasoning ability as the *outcome* and sought to identify traits associated with clinical reasoning. We examined the potential influence of *skill* risk literacy, as it is crucial for a higher general decision-making ability and decision-making styles, as they depict the *process* by which decisions are made. Lastly, medical students’ *motivations* towards a career in academia was observed, thereby making this the first study to research the aforementioned traits and their connections in aspiring physicians.

## Methods

### Setting and subjects

We conducted a prospective, non-randomized, cross-sectional study of regular third year medical students who were studying at the University of Münster in 2019. We chose students at this stage of their education as they had just completed the obligatory preliminary medical exam, which sets a base level of statistical knowledge.^1^ Data sets with missing answers, withdrawn participation, and incomplete attendance of the course were excluded, as it indicated the student to be of a higher semester, re-taking the course. Participation was anonymous and voluntary, and informed consent was obtained from all participants. Approval for this study was obtained from the ethics committee of the Chamber of Physicians at Westfalen-Lippe and the Medical Faculty of the University of Münster [2019–221-f-S].

### Study design

This study examined the participants’ (1) risk literacy, (2) decision-making styles, (3) their ability to solve Bayesian problems in medical case scenarios, and (4) and future medical career goals. For the construct of risk literacy, we used the Berlin Numeracy Test (BNT), which is a four-item, validated, psychometric tool used to evaluate a person’s risk literacy [13]. We chose it for its high discriminant and convergent validity and high predictive validity [13]. In addition, we used the Decision Styles Scale (DSS), introduced by Hamilton et al. [22], which centers on intuitive and rational decision-making. A clear factor structure and high internal consistency were exhibited across eight independent samples [22, 30]. The third set of questions contained medical case scenarios measuring Bayesian reasoning skills. The presented question format has been used before in studies among medical students and physicians [14–17]. Lastly, we surveyed medical students’ academic career plans with three self-created questions that closely depict medical students’ possible academic career paths in Germany. Furthermore, students were required to do mental arithmetic, as calculators were not allowed.

### Data collection

To objectify the required psychometrics, we distributed a paper-based questionnaire including four different segments: (1) the Berlin numeracy test (BNT), (2) the Decision styles scale (DSS), (3) three medical case scenarios (MCS), and (4) three questions concerning academic career goals. Firstly, subjects had to complete the Berlin numeracy test, which consists of four multiple choice questions testing for numeracy skills. Despite providing a selection of answers, this BNT format maintains balanced results [13]. Since its introduction and validation in 2012, it has been used in a number of studies with medical personnel or students as their target group [18, 31, 32]. Secondly, the DSS was administered. This instrument consists of ten items (five rational and five intuitive) aimed at assessing whether subjects lean towards intuitive or rational decision-making [22]. For the third segment, we constructed three medical case scenarios. The task in each scenario was to calculate the positive predictive value (PPV) of a diagnostic test using Bayes’ Theorem with the values of prevalence, sensitivity, and specificity. We constructed each medical case scenario to adhere to the following criteria:

- All scenarios must be solvable without a calculator. Therefore, to help mental arithmetic, every intermediate result should be an even number.
- The given data (prevalence, sensitivity, and specificity) should be as close to real data as possible to provide face validity for this segment.
- There must be variety in applying Bayes’ Theorem. In the first scenario, the participants were given sensitivity and specificity; in the second, sensitivity as well as 1-specificity; and in the third, 1-sensitivity and specificity.
- Students had to give exact estimates.
- The data were presented in the form of conditional probabilities.

In the last part, we asked students to rate their motivations toward academic career goals on a five-point Likert scale. The answers included achieving a doctorate degree, working in a university hospital, and conducting scientific research. In Germany, medical students can start working toward a doctorate degree before finishing their final exams. The question regarding conducting medical research was framed as working as a researcher either full-time or part-time in addition to clinical work. Thus, the latter question aimed to assess who was inclined toward continuous medical research and not only conducting research to achieve a doctorate degree.

### Outcome measures

As outcome measures, we separately quantified the total test scores of the BNT and the medical case scenarios. For the DSS, average scores on the rational and intuitive subscales were counted independently. In the last section regarding future career choices, subjects’ average scores in each category were measured.

### Statistical methods

To analyze the effect of possible influences on medical students’ Bayesian reasoning skills, we used an ordinal regression analysis and defined the score of the medical case scenarios as the outcome variable. We chose a cumulative model with the logistic link function which yields estimated log odds ratios as regression coefficients [33]. Our full model included all score variables of the three other test segments (BNT, DSS, and academic career goals) as well as age and gender. Despite their ordinal level, the score variables were treated as metric variables in the model. This was done due to the small number of participants combined with the unbalanced distribution of the outcome variable. These factors limited the number of regression coefficients that could be adequately estimated. Then, we performed a stepwise backward variable selection iteratively, excluding the variable with the highest *p*-value > 0.10 (likelihood ratio test). Results were reported as estimated odds ratios with corresponding 95% confidence intervals based on the profile likelihood [34] and p-values of the likelihood ratio test. To visualize the results, we plotted the conditional probabilities given the influencing variables of achieving a particular score in the medical case scenarios against each of the influencing variables at a time. The remaining influencing variables were fixed at the median. Ordinal logistic regression was modelled in R (version 4.1.2; R Core Team, 2022 [35]) with the MASS package [36]. The results were plotted with the ggplot2 package [37]. All analyses were exploratory for hypothesis generation and were not adjusted for multiple testing. *P*-values and confidence limits were two-sided and were intended to be exploratory, not confirmatory. In this exploratory sense, *p*-values ≤ 0.05 were considered statistically noticeable (“significant”).

## Results

### Descriptive data

**Fig. 1:**
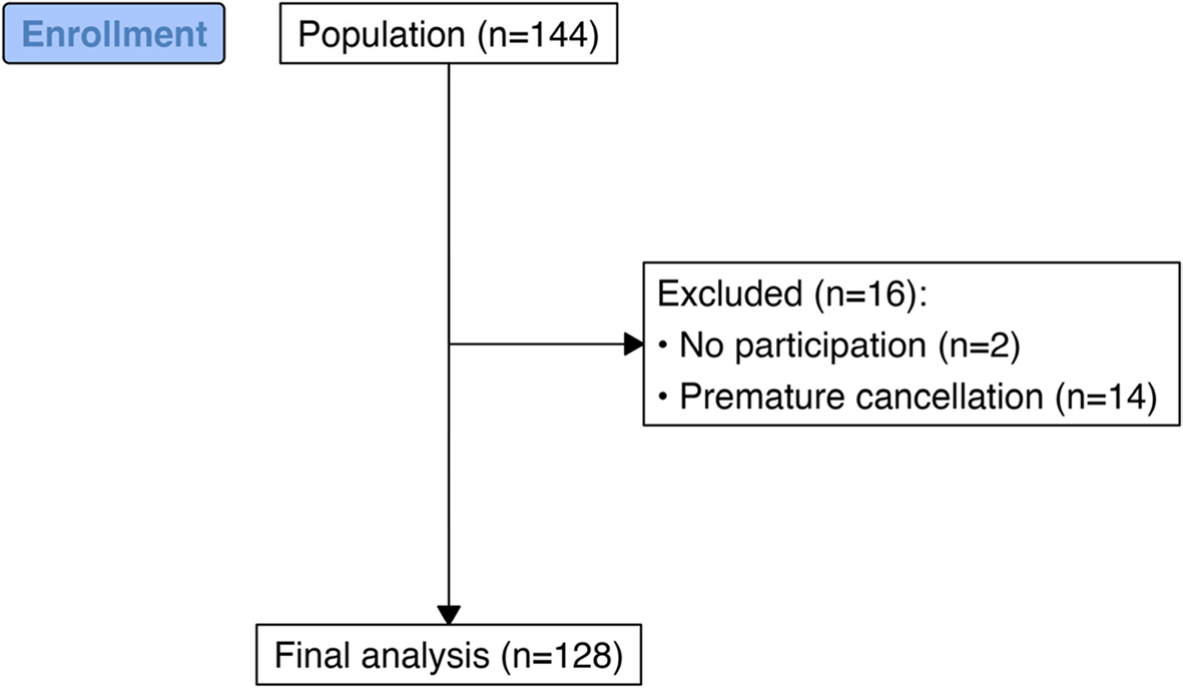
Exclusion process of questionnaires Premature cancellation refers to students who quit the study during the questionnaire

From 144 received questionnaires, we included 128 for further analysis. Reasons for exclusion of irregular participations or irregular datasets are shown in Fig. 1. This resulted in a study population with a mean age of 22.5 years (SD = 3.58) and a gender distribution with 68.5% women and 31.5% men (see Table 1). Descriptive data are summarized in Table 2. With a mean BNT score of 2.85 (SD = 1.08), the study population scored higher than the reference group (mean = 2.2) provided by Cokely et al. [13].

**Table 1:** Population characteristics

Demographic variables	*Mean*	*Median*	*SD*	*Min*	*Max*
Age	22.49	21	3.58	19	40
	*male*	*female*			
Gender	31.49%	68.51%			

Note. *SD* standard deviation, *Min* minimum, *Max* maximum

**Table 2:** Primary outcomes of three test segments

Test segment	*Mean*	*Median*	*SD*	*Min*	*Max*
Berlin Numeracy Test (BNT; 0 to 4 points)	2.85	3	1.08	0	4
Decision Styles Scale, rational subscale (DSS_rat; 0 to 5 points)	4.14	4.2	0.49	2.2	5
Decision Styles Scale, intuitive subscale (DSS_int; 0 to 5 points)	2.62	2.6	0.63	1	4
Medical case scenarios (0–3 points)	0.37	0	0.84	0	3
Future career plans (questionnaire with a 5-point Likert scales, 1–5)					
Doctorate degree	4.51	5	0.66	2	5
Medical research	2.63	2	1.22	1	5
Working in a University hospital	3.3	3	1.11	1	5

Note*. SD* standard deviation, *Min* minimum, *Max* maximum

Regarding decision-making styles, most students were far more inclined toward a rational decision style rather than intuitive decision-making. The quota of solved medical case examples was rather low, with only 25 out of 128 students scoring ≥ 1 point. Furthermore, in the prostate screening scenario, most students incorrectly chose the test’s specificity as the positive predictive value, showing that they were prone to a base-rate neglect. This can also be observed in physicians, regardless of their specialty [17]. From the three scenarios the abdominal pain scenario had the highest number of correct answers, while the chest pain scenario had the lowest one (see Table 3). Lastly, students exhibited unanimous readiness to achieve a doctorate degree (mean = 4.51 SD = 0.66), whereas ambitions of working in a university hospital or conducting medical research were more balanced.

**Table 3:** Three medical case scenarios with varying given variables and one target variable

Scenario	*Given Variables*	*Target Variable*	*Difficulty Index*
Chest pain	Prevalence, Sensitivity, Specificity	PPV	10.16%
Abdominal pain	Prevalence, Sensitivity, 1-Specificity	PPV	13.28%
Prostate cancer screening	Prevalence, 1-Sensitivity, Specificity	PPV	12.5%

Note. *PPV* positive predictive value, Difficulty Index = number of students who solved the medical case scenario

## Ordinal logistic regression

**Fig. 2:**
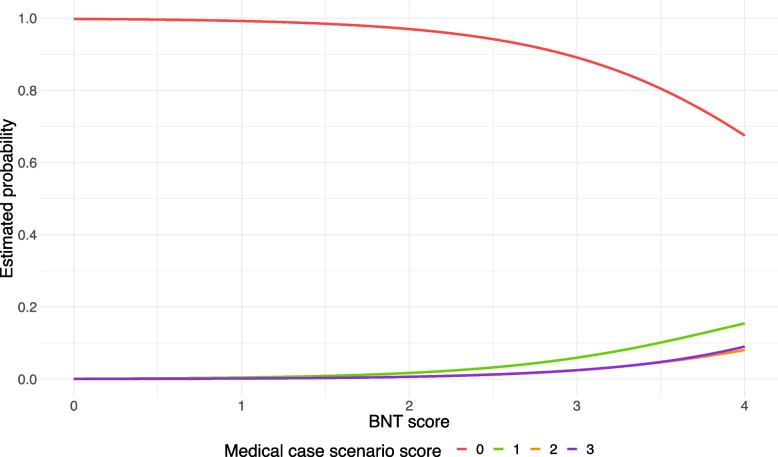
Estimated probabilities of achieving 0, 1, 2 and 3 points on the MCS, respectively, as a function of the BNT score, with ambitions to become a researcher fixed at the median score of 2

**Fig. 3:**
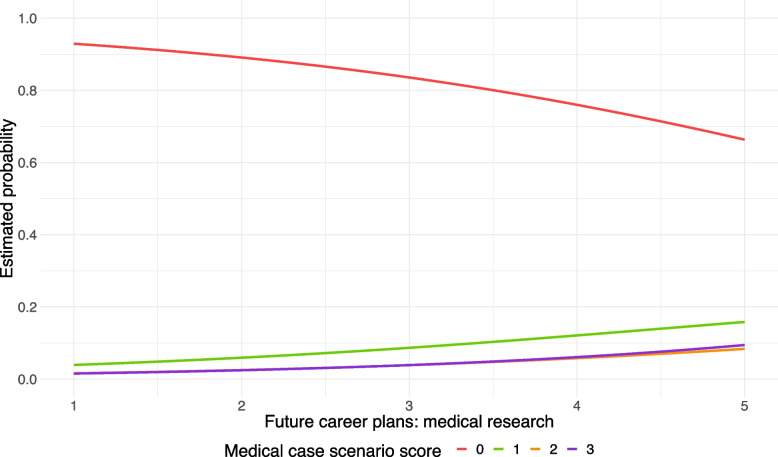
Estimated probabilities of achieving 0, 1, 2 and 3 points on the MCS, respectively, as a function of medical students’ ambitions to become a researcher, with the BNT score fixed at the median score of 3

**Fig. 4:**
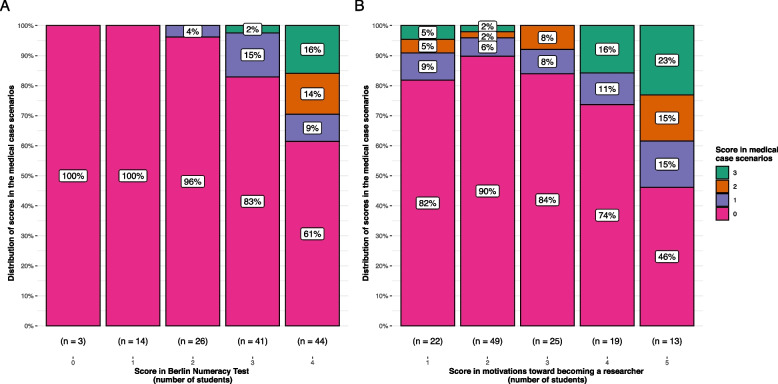
**A** distribution of medical case scenario scores for BNT scores. **B** Distribution of medical case scenario scores for scores on motivation towards becoming a researcher

Independent variable selection revealed only risk literacy (measured by the BNT) and inclination towards becoming a researcher to be related to Bayesian reasoning. Hence, decision-making styles and other career goals were excluded from the ordinal logistic regression model. Comparing the association of risk literacy and wanting to become a researcher with Bayesian reasoning skills, risk literacy was more important for solving Bayesian tasks. We observed a 33% increase in the chance of solving ≥ 1 medical case scenario when comparing the lowest BNT score to the highest (see Fig. 2). In contrast, only a 27% increase was seen in the chance of solving ≥ 1 medical case scenario when the lowest Likert score for motivations toward becoming a researcher was compared to the highest (see Fig. 3). The odds ratios demonstrated a similar trend (see Table 4). Moreover, there was a high increase in the probability of having ≥ 1 point in the medical case scenarios when the BNT score rose from three points to a maximum score of four (20% increase). This indicated that only highly risk literate medical students possessed the ability to obtain a positive predictive value in Bayesian tasks. Figure 3 contrasts this observation, as the Bayesian reasoning ability exhibited a more constant rise across the increasing motivation toward becoming a researcher. The same trend can be observed when comparing the distribution of the medical case scenarios scores for the BNT scores and the motivations to become a researcher (see Fig. 4).

**Table 4:** Explanatory variables influencing participants’ ability to solve Bayesian tasks (medical case scenarios) in an ordinal logistic regression model

Variables	Odds Ratio	95% CI		*p*
		*LL*	*UL*	
Berlin Numeracy Test	3.94	2.05	8.84	< 0.001
Inclination towards becoming a researcher	1.61	1.12	2.34	0.009

Note*.* Independent variable selection revealed only risk literacy (measured by the BNT) and inclination towards becoming a researcher to be related to Bayesian reasoning. Therefore, decision-making styles and other career goals were excluded from the OLR-model. *CI* confidence interval, *LL* lower limit, *UL* upper limit

## Discussion

This study aimed to link Bayesian reasoning, which represents parts of the diagnostic aspect of clinical reasoning, to medical students’ skills, decision process, and academic motivations. The results indicated that risk literacy and motivation toward becoming a researcher were related to Bayesian reasoning skills. Decision-making styles did not show statistically significant associations with better diagnostic judgement, indicating that this psychological construct might be too narrow and other processes such as the dual process framework might be more relevant to clinical reasoning [12]. Medical students preferred rational decision-making over intuitive decision-making, which might be because students at this level have not yet been able to encapsulate their pathophysiological knowledge into simplified causal models [38]. They would not yet be able to make fast judgements that feel like intuitive decision-making. The findings regarding risk literacy conform with prior research when general practitioners were selected as the study population [18]. With respect to non-medical personnel, available evidence is more conflicting. Ellis et al. [39] and Wegier and Shaffer [40] did not observe a relationship between numeracy and Bayesian reasoning. However, in a more recent study containing three samples of undergraduates, Brase [41] found numeracy to be a central trait for solving Bayesian tasks. In one out of their three experiments, Brase [41] demonstrated that thinking styles, which can be viewed as the broader construct encompassing decision-making styles, were statistically significantly linked with Bayesian reasoning. However, it was far less consistent and influential compared to numeracy. No further studies could be found linking medical students’ aspirations of becoming researchers and their Bayesian reasoning skills, making this the first study to do so. To our knowledge this is also the first study finding a positive association of risk literacy and clinical reasoning in medical undergraduate students, thereby identifying two new influences on clinical reasoning.

To contextualize the importance of risk literacy as a necessary skill for clinical reasoning, it is important to note how the effect of one influencing factor on a decision has much larger implications if that decision has to be made repeatedly [42]. To illustrate this statement, let us view these psychological constructs in a medical context. When attempting to reach a diagnosis, physicians are required to constantly update the likelihoods of differential diagnoses. This necessitates repeated decision-making under risk. These repeated decisions have a cumulative effect on the patient’s further treatment, as every evaluation of every examination, test, or information provided by the patient contributes to finding a diagnosis. Furthermore, physicians do not only see one patient but a myriad of them per shift. This means slight differences between physicians’ individual risk literacy have a large effect on their overall diagnostic accuracy. In a recent consensus statement the interpretation of such diagnostic tests has been identified as one of the key concepts of clinical reasoning that should be taught in medical schools [43]. Therefore, we can only lend support to the demand of nurturing numerical and statistical skills during medical studies [44–46], since it presents an underrepresented learning objective in medical curricula. Effective tools would be training interventions focusing on the application of visual aids and natural frequencies [54], as they are clearly helpful in solving Bayesian tasks [15, 47–51]. Contrary to risk literacy, the tendency toward a career in academia is not a skill which could be developed.

The above-mentioned cumulative effect would likely not occur in the relationship between Bayesian reasoning and motivation toward becoming a researcher. When considering this association, one should keep in mind that our study population was only in the third year out of a six-year degree program. Hence, their personal preferences could still change; therefore, we would cautiously reject the idea of a more meaningful association between these two factors. To make this assumption, the associated trait or ability should be stable over time, allowing it to have a constant influence [42]. A one-time survey of developing future physicians cannot provide this. Nonetheless, this indicates that scholarly motivations are a factor that can be associated with better clinical reasoning, opening up an opportunity to research what other motivations might be significant, since to our knowledge there is still a lack in studies investigating the influence of motivations on clinical reasoning.

## Limitations

The study’s first limitation could be that participants had not yet finished even half of their six-year degree program. Hence, their answers could still change in the future. This could be true for their decision-making styles and academic career goals. When it comes to risk literacy Friederichs et al. [31] found no statically significant increase in medical students throughout the years of university in a study population in which risk literacy had not been explicitly trained during their six-year training program. Bayesian reasoning skills are also unlikely to improve unless visual aids or natural frequencies are applied as above-mentioned studies of physicians demonstrate. Furthermore, the statistical explanatory power of this study is limited by the relatively low number of participants who were able to score ≥ 1 points in the medical case scenarios. This likely happened as a floor effect, which could also be observed in the results provided by Chapman and Liu [52]. In their study, almost none of the participants could correctly answer the Bayesian tasks; yet, when they offered the tasks in a natural frequency format, participants higher in numeracy profited from this. Therefore, presenting the Bayesian tasks as natural frequencies might have solved this problem. Lastly, this research is also limited by its observational design as it cannot imply actual causality.

## Conclusion

Physicians must be able to critically evaluate the implications of positive or negative test results to properly advise patients. The *skill* risk literacy and *motivations* toward becoming a researcher were identified as factors associated with clinical reasoning. Decision-making styles did not exhibit a significant association; however, other instruments measuring cognitive processes might produce different conclusions. Further research should be directed at assessing Bayesian reasoning skills in clinicians who conduct scientific work compared with clinicians who solely concentrate on clinical work. This would yield a study population where being a researcher would be a stable trait, providing more conclusive evidence. A similar study composition could be utilized for physicians with a doctorate degree and those who work in university hospitals with their counterparts.

## Supplementary Information

Supplementary Material 1.

## Data Availability

All data generated or analyzed during this study are included in this published article and its supplementary information files.

## Abbreviations

BNT: Berlin Numeracy Test
DSS: Decision styles scale
MCS: Medical case scenarios

## Footnotes

1 The catalogue containing the learning material (“Gegenstandskatalog”) provided by the IMPP (the official test administrators of the preliminary medical exam) includes methodological basics for survey conceptualisation and understanding of medical testing (prevalence, sensitivity, specificity, positive predictive value, and negative predictive value), e.g. having to compute a positive predictive value in a medical scenario [53].

## References

[1] Daniel M, Rencic J, Durning SJ, Holmboe E, Santen SA, Lang V, et al. Clinical Reasoning Assessment Methods: A Scoping Review and Practical Guidance. Acad Med. 2019;94(6):902–12. 10.1097/ACM.0000000000002618. 30720527 10.1097/ACM.0000000000002618

[2] Parodis I, Andersson L, Durning SJ, Hege I, Knez J, Kononowicz AA, et al. Clinical Reasoning Needs to Be Explicitly Addressed in Health Professions Curricula: Recommendations from a European Consortium. Int J Environ Res Public Health 2021;18(21). 10.3390/ijerph182111202. 10.3390/ijerph182111202 PMC8583438 34769721

[3] Young M, Thomas A, Gordon D, Gruppen L, Lubarsky S, Rencic J, et al. The terminology of clinical reasoning in health professions education: Implications and considerations. Med Teach. 2019;41(11):1277–84. 10.1080/0142159X.2019.1635686. 31314612 10.1080/0142159X.2019.1635686

[4] Young ME, Thomas A, Lubarsky S, Gordon D, Gruppen LD, Rencic J, et al. Mapping clinical reasoning literature across the health professions: a scoping review. BMC Med Educ. 2020;20(1):107. 10.1186/s12909-020-02012-9. 32264895 10.1186/s12909-020-02012-9 PMC7140328

[5] Pelaccia T, Plotnick LH, Audétat M-C, Nendaz M, Lubarsky S, Torabi N, et al. A Scoping Review of Physicians’ Clinical Reasoning in Emergency Departments. Ann Emerg Med. 2020;75(2):206–17. 10.1016/j.annemergmed.2019.06.023. 31474478 10.1016/j.annemergmed.2019.06.023

[6] Holdar U, Wallin L, Heiwe S. Why do we do as we do? Factors influencing clinical reasoning and decision-making among physiotherapists in an acute setting. Physiother Res Int. 2013;18(4):220–9. 10.1002/pri.1551. 23637022 10.1002/pri.1551

[7] Merisier S, Larue C, Boyer L. How does questioning influence nursing students’ clinical reasoning in problem-based learning? A scoping review Nurse Educ Today. 2018;65:108–15. 10.1016/j.nedt.2018.03.006. 29550674 10.1016/j.nedt.2018.03.006

[8] Andersson U, Maurin Söderholm H, Wireklint Sundström B, Andersson Hagiwara M, Andersson H. Clinical reasoning in the emergency medical services: an integrative review. Scand J Trauma Resusc Emerg Med. 2019;27:76. 10.1186/s13049-019-0646-y. 31426839 10.1186/s13049-019-0646-y PMC6700770

[9] Rencic J. Twelve tips for teaching expertise in clinical reasoning. Med Teach. 2011;33(11):887–92. 10.3109/0142159X.2011.558142. 21711217 10.3109/0142159X.2011.558142

[10] Bours MJ. Bayes’ rule in diagnosis. J Clin Epidemiol. 2021;131:158–60. 10.1016/j.jclinepi.2020.12.021. 33741123 10.1016/j.jclinepi.2020.12.021

[11] Elstein AS, Schwartz A, Schwarz A. Clinical problem solving and diagnostic decision making: selective review of the cognitive literature. BMJ. 2002;324(7339):729–32. 10.1136/bmj.324.7339.729. 11909793 10.1136/bmj.324.7339.729 PMC1122649

[12] Yazdani S, Hoseini AM. Five decades of research and theorization on clinical reasoning: a critical review. Adv Med Educ Pract. 2019;10:703–16. 10.2147/AMEP.S213492. 31695548 10.2147/AMEP.S213492 PMC6717718

[13] Cokely ET, Galesic M, Schulz E, Ghazal S, Garcia-Retamero R. Measuring Risk Literacy: The Berlin Numeracy Test. Judgement and Decision Making. 2012;7:25–47. 10.1037/t45862-000.

[14] Noguchi Y, Matsui K, Imura H, Kiyota M, Fukui T. Quantitative Evaluation of the Diagnostic Thinking Process in Medical Students. J Gen Intern Med. 2002;17(11):848–53. 10.1046/j.1525-1497.2002.20139.x. 10.1046/j.1525-1497.2002.20139.x PMC1495132 12406355

[15] Friederichs H, Ligges S, Weissenstein A. Using tree diagrams without numerical values in addition to relative numbers improves students’ numeracy skills: a randomized study in medical education. Med Decis Making. 2014;34(2):253–7. 10.1177/0272989X13504499. 24085290 10.1177/0272989X13504499

[16] Ghosh AK, Ghosh K, Erwin PJ. Do medical students and physicians understand probability? QJM. 2004;97(1):53–5. 10.1093/qjmed/hch010. 14702512 10.1093/qjmed/hch010

[17] Agoritsas T, Courvoisier DS, Combescure C, Deom M, Perneger TV. Does prevalence matter to physicians in estimating post-test probability of disease? A randomized trial. J Gen Intern Med. 2011;26(4):373–8. 10.1007/s11606-010-1540-5. 21053091 10.1007/s11606-010-1540-5 PMC3055966

[18] Friederichs H, Birkenstein R, Becker JC, Marschall B, Weissenstein A. Risk literacy assessment of general practitioners and medical students using the Berlin Numeracy Test. BMC Fam Pract. 2020;21(1):143. 10.1186/s12875-020-01214-w. 32664885 10.1186/s12875-020-01214-w PMC7362657

[19] Cokely ET, Feltz A, Ghazal S, Allan JN, Petrova D, Garcia-Retamero R. Skilled Decision Theory: From Intelligence to Numeracy and Expertise. In: Ericsson KA, Hoffman RR, Kozbelt A, Williams AM, editors. The Cambridge Handbook of Expertise and Expert Performance: Cambridge University Press; 2018. p. 476–505. 10.1017/9781316480748.026.

[20] Ghazal S, Cokely ET, Garcia-Retamero R. Predicting biases in very highly educated samples: Numeracy and metacognition. Judgm Decis Mak. 2014;9(1):15–34. 10.1017/S1930297500004952.

[21] Harren VA. A model of career decision making for college students. J Vocat Behav. 1979;14(2):119–33. 10.1016/0001-8791(79)90065-4.

[22] Hamilton K, Shih S-I, Mohammed S. The Development and Validation of the Rational and Intuitive Decision Styles Scale. J Pers Assess. 2016;98(5):523–35. 10.1080/00223891.2015.1132426. 26967981 10.1080/00223891.2015.1132426

[23] Scott SG, Bruce RA. Decision-Making Style: The Development and Assessment of a New Measure. Educ Psychol Measur. 1995;55(5):818–31. 10.1177/0013164495055005017.

[24] Cate O ten, Durning SJ. Understanding Clinical Reasoning from Multiple Perspectives: A Conceptual and Theoretical Overview. In: Cate O ten, Custers E, Durning S, editors. Principles and Practice of Case-Based Clinical Reasoning Education: A Method for Preclinical Students. New York: Springer. 2017. p. 35–46. 10.1007/978-3-319-64828-6_3.

[25] Phillips WJ, Fletcher JM, Marks ADG, Hine DW. Thinking styles and decision making: A meta-analysis. Psychol Bull. 2016;142(3):260–90. 10.1037/bul0000027. 26436538 10.1037/bul0000027

[26] The Royal College of Physicians and Surgeons of Canada. CanMEDS Role: Scholar. 10.06.2023. https://www.royalcollege.ca/ca/en/canmeds/canmeds-framework/canmeds-role-scholar.html. Accessed 10 Jun 2023.

[27] Gravanis MB. Characteristics of the Good Researcher: Innate Talent or Acquired Skills? Clin Cardiol. 2007;30(2):52–3. 10.1002/clc.24. 16532719

[28] Toledo-Pereyra LH. Ten qualities of a good researcher. J Invest Surg. 2012;25(4):201–2. 10.3109/08941939.2012.701543. 22853811 10.3109/08941939.2012.701543

[29] Provenzale D. A guide for success as a clinical investigator. Gastroenterology. 2012;142(3):418–21. 10.1053/j.gastro.2012.01.009. 22266151 10.1053/j.gastro.2012.01.009

[30] Hamilton K, Shih S-I, Mohammed S. The predictive validity of the decision styles scale: An evaluation across task types. Personality Individ Differ. 2017;119:333–40. 10.1016/j.paid.2017.08.009.

[31] Friederichs H, Schölling M, Marschall B, Weissenstein A. Assessment of Risk Literacy Among German Medical Students: A Cross-Sectional Study Evaluating Numeracy Skills. Hum Ecol Risk Assess Int J. 2014;20(4):1139–47. 10.1080/10807039.2013.821909.

[32] Garcia-Retamero R, Cokely ET, Wicki B, Joeris A. Improving risk literacy in surgeons. Patient Educ Couns. 2016;99(7):1156–61. 10.1016/j.pec.2016.01.013. 26879804 10.1016/j.pec.2016.01.013

[33] Bürkner P-C, Vuorre M. Ordinal Regression Models in Psychology: A Tutorial. Adv Methods Pract Psychol Sci. 2019;2(1):77–101. 10.1177/2515245918823199.

[34] Minkin S, Venzon DJ. Profile-likelihood-based confidence intervals. Reply. Appl Stat. 1990;39:125–7.

[35] R Core Team. R: A language and environment for statistical computing. Vienna, Austria: R Foundation for Statistical Computing; 2022.

[36] Venables WN, Ripley BD. Modern applied statistics with S. 4th ed. New York: Springer; 2002.

[37] Wickham H. ggplot2: Elegant Graphics for Data Analysis. 2^nd^ ed. Cham: Springer International Publishing; Imprint: Springer; 2016.

[38] Schmidt HG, Rikers RMJP. How expertise develops in medicine: knowledge encapsulation and illness script formation. Med Educ. 2007;41(12):1133–9. 10.1111/j.1365-2923.2007.02915.x. 18004989 10.1111/j.1365-2923.2007.02915.x

[39] Ellis KM, Cokely ET, Ghazal S, Garcia-Retamero R. Do People Understand their Home HIV Test Results? Risk Literacy and Information Search. Proceedings of the Human Factors and Ergonomics Society Annual Meeting. 2014;58(1):1323–7. 10.1177/1541931214581276.

[40] Wegier P, Shaffer VA. Aiding risk information learning through simulated experience (ARISE): Using simulated outcomes to improve understanding of conditional probabilities in prenatal Down syndrome screening. Patient Educ Couns. 2017;100(10):1882–9. 10.1016/j.pec.2017.04.016. 28526191 10.1016/j.pec.2017.04.016

[41] Brase G. Which cognitive individual differences predict good Bayesian reasoning? Concurrent comparisons of underlying abilities. Mem Cognit. 2021;49(2):235–48. 10.3758/s13421-020-01087-5. 10.3758/s13421-020-01087-5 32815106

[42] Funder DC, Ozer DJ. Evaluating Effect Size in Psychological Research: Sense and Nonsense. Adv Methods Pract Psychol Sci. 2019;2(2):156–68. 10.1177/2515245919847202.

[43] Cooper N, Bartlett M, Gay S, Hammond A, Lillicrap M, Matthan J, Singh M. Consensus statement on the content of clinical reasoning curricula in undergraduate medical education. Med Teach. 2021;43(2):152–9. 10.1080/0142159X.2020.1842343. 33205693 10.1080/0142159X.2020.1842343

[44] Rao G. Physician numeracy: essential skills for practicing evidence-based medicine. Fam Med. 2008;40(5):354–8. 18465286

[45] Hanoch Y, Miron-Shatz T, Cole H, Himmelstein M, Federman AD. Choice, numeracy, and physicians-in-training performance: the case of Medicare Part D. Health Psychol. 2010;29(4):454–9. 10.1037/a0019881. 20658834 10.1037/a0019881 PMC5605761

[46] Wegwarth O, Gigerenzer G. The Barrier to Informed Choice in Cancer Screening: Statistical Illiteracy in Physicians and Patients. Recent Results Cancer Res. 2018;210:207–21. 10.1007/978-3-319-64310-6_13. 28924688 10.1007/978-3-319-64310-6_13

[47] McDowell M, Jacobs P. Meta-analysis of the effect of natural frequencies on Bayesian reasoning. Psychol Bull. 2017;143(12):1273–312. 10.1037/bul0000126. 29048176 10.1037/bul0000126

[48] Hoffrage U, Gigerenzer G. Using natural frequencies to improve diagnostic inferences. Acad Med. 1998;73(5):538–40. 9609869 10.1097/00001888-199805000-00024

[49] Binder K, Krauss S, Schmidmaier R, Braun LT. Natural frequency trees improve diagnostic efficiency in Bayesian reasoning. Adv Health Sci Educ Theory Pract. 2021;26(3):847–63. 10.1007/s10459-020-10025-8. 33599875 10.1007/s10459-020-10025-8 PMC8338842

[50] Galesic M, Gigerenzer G, Straubinger N. Natural frequencies help older adults and people with low numeracy to evaluate medical screening tests. Med Decis Making. 2009;29(3):368–71. 10.1177/0272989X08329463. 19129155 10.1177/0272989X08329463

[51] Talboy AN, Schneider SL. Improving Understanding of Diagnostic Test Outcomes. Med Decis Making. 2018;38(5):573–83. 10.1177/0272989X18758293. 29608866 10.1177/0272989X18758293

[52] Chapman GB, Liu J. Numeracy, frequency, and Bayesian reasoning. Judgm decis mak. 2009;4(1):34–40. 10.1017/S1930297500000681.

[53] Institut für Medizinische und Pharmazeutische Prüfungsfragen [Institute for Medical and Pharmaceutical testing]. IMPP-Gegenstandskatalog (IMPP-GK 1) für den schriftlichen Teil des ersten Abschnitts der ärztlichen Prüfung (ÄAppO vom 27. Juni 2002): Teilkatalog „Grundlagen der Medizinischen Psychologie und der Medizinischen Soziologie“ [IMPP-catalog of learning material (IMPP-GK 1) for the written part of the first section of the physician exam (ÄAppO 27th of June 2002): part of the catalog „Basics of Medical Psychology and Sociology“]. 2010. https://www.impp.de/pruefungen/allgemein/gegenstandskataloge.html. German. Accessed 13 Apr 2025.

[54] Jenny MA, Keller N, Gigerenzer G. Assessing minimal medical statistical literacy using the Quick Risk Test: a prospective observational study in Germany. BMJ Open. 2018;8(8): e020847. 10.1136/bmjopen-2017-020847. 10.1136/bmjopen-2017-020847 PMC6112405 30139896

